# Off-Nadir Hyperspectral Sensing for Estimation of Vertical Profile of Leaf Chlorophyll Content within Wheat Canopies

**DOI:** 10.3390/s17122711

**Published:** 2017-11-23

**Authors:** Weiping Kong, Wenjiang Huang, Raffaele Casa, Xianfeng Zhou, Huichun Ye, Yingying Dong

**Affiliations:** 1 Key Laboratory of Digital Earth Science, Institute of Remote Sensing and Digital Earth, Chinese Academy of Sciences, Beijing 100094, China; kongwp@radi.ac.cn (W.K.); zhouxf@radi.ac.cn (X.Z.); yehuichun000@126.com (H.Y.); dongyy@radi.ac.cn (Y.D.); 2 University of Chinese Academy of Sciences, Beijing 100049, China; 3 State Key Laboratory of Remote Sensing Science, Institute of Remote Sensing and Digital Earth, Chinese Academy of Sciences, Beijing 100094, China; 4 Department of Agricultural and Forestry scieNcEs (DAFNE), Università degli Studi della Tuscia, Via San Camillo de Lellis, 01100 Viterbo, Italy; rcasa@unitus.it

**Keywords:** off-nadir spectral reflectance, vertical profile, leaf chlorophyll content, optimized spectral indices, winter wheat

## Abstract

Monitoring the vertical profile of leaf chlorophyll (Chl) content within winter wheat canopies is of significant importance for revealing the real nutritional status of the crop. Information on the vertical profile of Chl content is not accessible to nadir-viewing remote or proximal sensing. Off-nadir or multi-angle sensing would provide effective means to detect leaf Chl content in different vertical layers. However, adequate information on the selection of sensitive spectral bands and spectral index formulas for vertical leaf Chl content estimation is not yet available. In this study, all possible two-band and three-band combinations over spectral bands in normalized difference vegetation index (NDVI)-, simple ratio (SR)- and chlorophyll index (CI)-like types of indices at different viewing angles were calculated and assessed for their capability of estimating leaf Chl for three vertical layers of wheat canopies. The vertical profiles of Chl showed top-down declining trends and the patterns of band combinations sensitive to leaf Chl content varied among different vertical layers. Results indicated that the combinations of green band (520 nm) with NIR bands were efficient in estimating upper leaf Chl content, whereas the red edge (695 nm) paired with NIR bands were dominant in quantifying leaf Chl in the lower layers. Correlations between published spectral indices and all NDVI-, SR- and CI-like types of indices and vertical distribution of Chl content showed that reflectance measured from 50°, 30° and 20° backscattering viewing angles were the most promising to obtain information on leaf Chl in the upper-, middle-, and bottom-layer, respectively. Three types of optimized spectral indices improved the accuracy for vertical leaf Chl content estimation. The optimized three-band CI-like index performed the best in the estimation of vertical distribution of leaf Chl content, with R^2^ of 0.84–0.69, and RMSE of 5.37–5.56 µg/cm^2^ from the top to the bottom layers, while the optimized SR-like index was recommended for the bottom Chl estimation due to its simple and universal form. We suggest that it is necessary to take into account the penetration characteristic of the light inside the canopy for different Chl absorption regions of the spectrum and the formula used to derive spectral index when estimating the vertical profile of leaf Chl content using off-nadir hyperspectral data.

## 1. Introduction

Dynamic monitoring of chlorophyll (Chl) content of crop plants during different growth stages provides crucial information to understand plants’ physiological status and their response to environmental changes in agro-ecosystems [1,2]. Indeed, leaf Chl, as the main photosynthetic pigment, can be directly related to leaf nitrogen status, plant stress, and senescence [3,4]. Crop canopies generally exhibit vertical heterogeneity of leaf Chl because of different light environment, growth and development of the plant itself, nitrogen uptake and remobilization processes [5]. Therefore, monitoring the vertical distribution of leaf Chl content within a canopy would be useful to reveal actual crop growth conditions and nutritional status. An increasing number of relevant studies have been carried out from the perspective of plant physiology for different crops, such as wheat, maize, soybean, sunflower, etc. [2,5,6,7]. Since the lower leaves of the canopy are more prone to nutrient deficits and plant senescence than the upper leaves [8], knowledge of leaf Chl content in the lower layers has significant interest, e.g., in precision agriculture. It would allow the early detection of subtle variations within the field, rather than taking steps after crop stress has happened.

The development of remote sensing techniques has enabled us to estimate crop Chl content at the field or regional scales. Numerous studies have focused on the remote estimation of crop Chl content by means of physical, empirical and semi-empirical methods [1,9,10,11,12]. Partial or even full spectral information was employed, but the practical use of such approaches would be limited, given the current infrequent availability of hyperspectral data [13], especially for spaceborne sensors. Spectral indices have been proposed, using spectral reflectance measurements most often acquired from a near nadir position. They were derived from two or more spectral wavebands according to mathematical formulas, in order to maximize the sensitivity to leaf Chl. Two-band spectral combinations, calculated as a normalized difference vegetation index (NDVI) or a simple ratio (SR), are the most widely used, both for ground based [14] and airborne or satellite applications [9,15]. Nevertheless, they utilize only a limited amount of information available in spectral data [16,17] and are susceptible to saturation. As a better alternative, three-band spectral indices have been proposed [18,19]. However, most of these studies rarely took into account the heterogeneous distribution of leaf Chl content, thereby possibly resulting in errors in the estimation of overall canopy Chl content and of the related crop nitrogen status [5,7,20].

In recent years, researchers have attempted to explore new remote sensing approaches for quantifying vertical leaf Chl distribution within crop canopies [21,22,23]. Whereas the nadir downward viewing spectral reflectance contains information mainly from the upper canopy layer [20], off-nadir observations could provide more information on the full extent of canopy depth. Huang et al. [21] conducted a great deal of work studying the leaf Chl content in different vertical layers and proposed a spectral index derived from ground-based multi-angle hyperspectral reflectance. Their results provide essential knowledge to estimate vertical distribution of leaf Chl content. Currently, tractor-based proximal sensors are available on the market and some of them have off-nadir viewing angles setups [24]. These measurement configurations offer an advantage as compared to nadir looking sensors since they avoid the use of sensor booms, which might create their own shadows, and are less affected by soil conditions. However, in applying this technology, potentially sensitive to vertical leaf Chl information, the underlying mechanisms of multiple scattering inside plant canopies has not been adequately taken into account. It is well known that spectral light composition changes vertically within a canopy, becoming gradually less rich in the blue to red bands due to selective leaf absorption [25]. In addition, bands in the red edge region have been shown, using physically-based radiative transfer models, to have much higher penetration depth than green light inside the canopy [26]. Bands at longer wavelengths, e.g., in the SWIR, have an even higher canopy penetration, though they are not sensitive to chlorophyll. It is therefore logical to expect that different spectral band combinations might provide better relationships to leaf pigments, for the different layers of the canopy, as compared to a single band combination. From a practical point of view, it is also important to identify which is the best angle that can capture the maximum spectral information for a specific leaf layer and to investigate how spectral band combinations and different formulas of spectral indices impact the estimation of vertical leaf Chl content. Some studies showed changes of relationships between published nadir-proposed spectral indices and leaf Chl content of the whole canopy at different viewing angles [27,28]. Nevertheless, few researches have reported the changes in sensitivity of these indices to leaf Chl content in vertical layers. Given the vertical heterogeneity of Chl content and leaf stacking effect within the canopy, is it accurate enough to estimate leaf Chl in different layers by using the same spectral index?

The aim of this study was to systematically investigate the selection of sensitive band combinations in spectral indices in order to identify the best performing spectral indices for remotely estimating leaf Chl content in different vertical layers of winter wheat canopies. The specific objectives were to: (i) analyze how leaf Chl content is vertically distributed in winter wheat at different growth stages; (ii) illustrate the response characteristics of hyperspectral reflectance at different viewing angles; (iii) discuss the sensitivity of published spectral indices to vertical leaf Chl content at different viewing angles and identify the types of reliable two- and three-band spectral indices for leaf Chl estimation; and (iv) optimize the band combinations of two- and three-band spectral indices at different viewing angles and compare their performances for the quantification of the vertical profile of leaf Chl content.

## 2. Materials and Methods

### 2.1. Study Site

The study site was located at the National Experiment Station for Precision Agriculture (40°10.6′ N, 116°26.3′ E), Beijing, China. This area has been operational since 2001 and used for precision agriculture research, with a mean annual rainfall of 507.7 mm and a mean annual temperature of 13.8 °C. The soil is silty clay loam, with nutrient content in the topsoil layer (0–0.20 m depth) about 1.42–2.2% of organic matter, 117.6–129.1 mg/kg of available potassium, and 20.1–55.4 mg/kg of available phosphorus. Winter wheat is one of the most important crops in China and was selected for this study. The field experiments were conducted in the period 2005–2007 using eleven erectophile-type cultivars: Jing411, Nongda3291, 9158, Jingdong12, Laizhou3279, I-93, 6211, Jing9843, Lumai21, P7, and Xiaoyan54. All cultivars were sown with a density of 3 × 10^6^ plants ha^−1^ and a row spacing of 25 cm. Each cultivar was planted in a plot with an area of 45 × 10.8 m^2^.

Spectral measurements and leaf Chl determinations were carried out at typical winter wheat growth stages: 24 April (Stem elongation, Z34), 10 May (Booting, Z47), and 20 May (Heading, Z59) 2005; 20 May (Heading, Z59) and 30 May (Milk-filling, Z73) 2006; and 17 April (Stem elongation, Z31), 28 April (Stem elongation, Z39), 9 May (Booting, Z47), 19 May (Heading, Z59) and 29 May (Milk-filling, Z73) 2007. A total of sixty-seven samples were collected among the three years and distributed as follows: 18, 4 and 45 in 2005 to 2007, respectively.

### 2.2. Multi-Angle Hyperspectral Reflectance Measurement

An ASD FieldSpec 3 spectrometer (Analytical Spectral Devices, Boulder, CO, USA), with a 25° field-of-view fiber optics, was used to measure canopy spectral reflectance under clear sky conditions between 11:00 a.m. and 13:00 p.m. (Beijing local time), when minimum variations in solar view angle occur. A rotating bracket was used to hold the instrument, enabling spectral measurements of the same target from different angles in a short time. The observations were made in the principal plane (constructed by the direction of incident direct sun light, and the direction of the normal to surface target) at different viewing zenith angles (VZAs). The nadir (i.e., observation zenith angle 0°) spectral measurements were made at a height of 1 m above canopy top. The off-nadir spectral measurements were made from −60° to +60° with 10° incremental steps, where a positive angle corresponds to backscattering direction (measured back to the sun) and a negative angle corresponds to forward scattering direction (measured face to the sun). Each spectral measurement was preceded by a dark current measurement and a white reference measurement using a white Spectralon^®^ (Labsphere, Inc., North Sutton, NH, USA) reference panel. More detailed information about the multi-angle spectral measurements can be found in previous studies [21,29]. Twenty scans were performed and averaged to obtain spectral reflectance for each observing direction.

### 2.3. Leaf Chlorophyll Content Vertical Distribution Measurement

**Figure 1:**
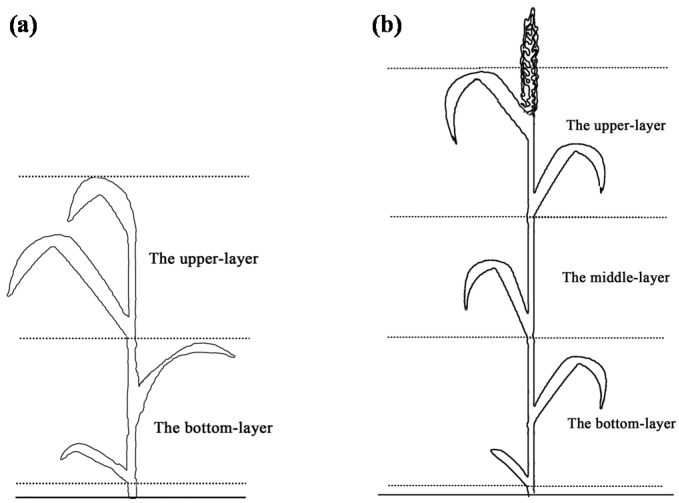
The schematic diagram of the divisions of: two layers at the stem elongation stage (Z31) stage (**a**); and three layers at the other growth stages (**b**) in wheat canopies.

Wheat samples were collected in the 1 m × 1 m area encompassing the canopy where spectra had been acquired. They were harvested and immediately brought to the laboratory after multi-angle spectral measurements. Plant leaves were cut off and divided into three layers according to their positions within the canopy (Figure 1): the top 1st and 2nd leaves were assigned to the upper-layer, the top 3rd leaves to the middle-layer, the top 4th and subsequent leaves below were assigned to the bottom-layer. Leaves sampled at the stem elongation stage (Z31) were divided only into the upper and bottom layers, owing to the limited number of leaves at that stage. Leaf Chl content in each layer were determined separately. 

Two leaf disks of 0.25 cm^2^ were cut off from each leaf sample belonging to the same layer. One of the disks was used for the determination of dry weight (DW, g), which was weighted after drying the samples in an oven at 75 °C for 48 h. The other was used for the extraction of pigments, which was carried out by immersing and grinding the disk in 10 mL aqueous acetone/distilled water buffer solution (80:20, volume proportion). After storing the solution in darkness for more than 24 h, the absorbance was measured with a UV-VIS spectrophotometer (Perkin-Elmer, Lambda 5, Waltham, MA, USA) at 645, 663 and 470 nm wavelengths. Chl a, Chl b and carotenoid concentration (mg/L) were calculated using specific absorption coefficients and the absorbance measured at 645, 663 and 470 nm with Equations (1)–(3) [30]. Then, the unit of Chla and Chlb concentration was converted into mg/g, i.e., mass per unit leaf dry weight, with Equations (4) and (5). Leaf Chl content (µg/cm^2^), used as a target variable in this study, was calculated by Equation (6).

Chla(mg/L) = 12.25 A_663_ − 2.79 A_645_ (1)

Chlb(mg/L) = 21.50 A_645_ − 5.10 A_663_ (2)

Carotenoid(mg/L) = (1000 A_470_ − 1.82 Chla −85.02 Chlb)/198 (3)

Chla(mg/g) = [Chla(mg/L) × V_T_(mL)]/[DW(g) × 1000] (4)

Chlb(mg/g) = [Chlb(mg/L) × V_T_(mL)]/[DW(g) × 1000] (5)

Chl(µg/cm^2^) = [(Chla(mg/g) + Chlb(mg/g)) × DW(g) × 1000]/leaf area(cm^2^) (6)

where A_645_, A_663_ and A_470_ are the absorbance of extract solution at wavelength 645, 663 and 470 nm respectively; V_T_(mL) is the volume of extract solution; leaf area(cm^2^) is the total area of the leaf disks used for leaf DW measurements.

### 2.4. Vegetation Indices and Data Analysis

#### 2.4.1. Published Vegetation Indices

A set of published Chl-related spectral indices were used and classified into two categories: two-band spectral indices and three-band spectral indices (Table 1).

#### 2.4.2. Computing Two-Band and Three-Band Spectral Indices

All possible two-band combinations (*λ*1 and *λ*2) from 400 to 1000 nm in SR and NDVI types of indices, referred to as SR-like and NDVI-like indices, were calculated at different VZAs. A three-band chlorophyll index (CI), proposed by Gitelson et al. [39], has been applied successfully to determine Chl content for different crop species. We used CI-like type of indices, calculated from all possible three-band combinations (*λ*1, *λ*2 and *λ*3) in the regions of the green to red bands (470–730 nm), the red to red edge bands (680–800 nm) and the NIR bands (740–1000 nm). The three types of spectral indices were defined as Equations (7)–(9). They were used to optimize the most sensitive two-band and three-band combinations for estimating leaf Chl content in each vertical layer, referred to as the Optimized SR-like(*λ*1, *λ*2), Optimized NDVI-like(*λ*1, *λ*2) and Optimized CI-like(*λ*1, *λ*2, *λ*3). All calculations were implemented using MATLAB 8.3 (The MathWorks, Inc., Natick, MA, USA).

(7) $$\text{SR-like}(\lambda 1,\;\lambda 2)=\frac{R_{\lambda 1}}{R_{\lambda 2}}$$

(8) $$\text{NDVI-like}(\lambda 1,\;\lambda 2)=\frac{R_{\lambda 1}-R_{\lambda 2}}{R_{\lambda 1}+R_{\lambda 2}}$$

(9) $$\text{CI-like}(\lambda 1,\;\lambda 2,\;\lambda 3)=(R_{\lambda 1}^{-1}-R_{\lambda 2}^{-1})\times R_{\lambda 3}$$

It should be noted that spectral reflectance obtained at different VZAs were processed and analyzed as an individual dataset in our study.

#### 2.4.3. Model Calibration and Validation

Altogether, 67 wheat samples were collected in 2005, 2006 and 2007. Since our data included different growth stages of wheat among the three years, samples in each growth stage in each year were randomly divided into two datasets: two thirds as the training dataset and one third as the validation dataset. Thus, 45 samples were used to build estimation models and the remaining 22 samples were used to validate leaf Chl prediction for the upper and bottom layers of the canopy. For the middle-layer, 38 samples were used for calibration and 20 for validation, because of limited leaves within wheat canopy at the stem elongation stage (Z31) in 2007.

Linear regression was used to model the relationship between published and optimized spectral indices and leaf Chl in vertical layers at different VZAs and to validate the models. To compare the performance of spectral indices, we calculated coefficients of determination (R^2^), *p*-value and root mean square error (RMSE). The higher R^2^ and the lower RMSE, the better is the accuracy of the index. In addition, the relative root mean square error (RRMSE) was employed to compare different variables or different ranges, being less sensitive to the absolute values [41]. It was used in the comparison of spectral indices among different vertical layers. RMSE and RRMSE were calculated as follows:

(10) $$\mathrm{RMSE}=\sqrt{\sum_{i=1}^{n}{\left({\hat{y}}_{i}-y_{i}\right)}^{2}/n}$$

(11) $$\mathrm{RRMSE}=100\times \mathrm{RMSE}/{\bar{y}}_{i}$$

where ${\hat{y}}_{i}$ and $y_{i}$ are the estimated and measured leaf Chl content, respectively; ${\bar{y}}_{i}$ is the mean of the measured leaf Chl content; and *n* is the number of samples. RRMSE < 10% is considered as excellent model performance, while 10% < RRMSE < 20% is considered as good model performance [41].

## 3. Results

### 3.1. Vertical Distribution of Leaf Chlorophyll Content within Wheat Canopy

**Figure 2:**
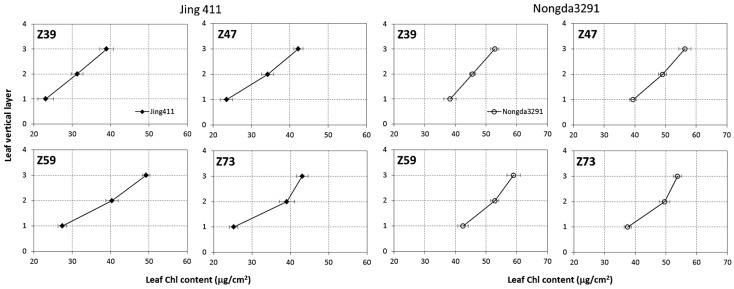
Vertical distribution of leaf Chl content within wheat canopies measured in 2007 during stem elongation (Z39), booting (Z47), heading (Z59) and milk-filling (Z73) stages for: Jing411 (**left**); and Nongda3291 (**right**) cultivars. The values 3, 2, and 1 on *y*-axis indicate the upper-, middle- and bottom-layer, respectively. Standard deviation of measurements is drawn as horizontal error bars.

The vertical profile of leaf Chl content within wheat canopies during different growth stages is presented in Figure 2. Jing411 and Nongda3291, two dominant and widely grown cultivars in the North China Plain [42], were chosen as examples. Their leaf colors are light green and dark green, respectively. As shown in Figure 2, leaf Chl content showed in general a decreasing trend from top to bottom at each growth stage. The differences of leaf Chl between the middle- and bottom-layer were much larger than those between the upper- and middle-layer, after the stem elongation stage (Z39). This was probably due to potential localized leaf nutrition deficiencies caused by nitrogen remobilization in plant from old leaves to new leaves [43], as well as leaf senescence in the lower layer. During the whole growth stages, the changing patterns of leaf Chl content in all three layers of the two cultivars were similar, with the highest values at the heading stage (Z59).

### 3.2. Response Characteristics of Spectral Reflectance among Different VZAs

**Figure 3:**
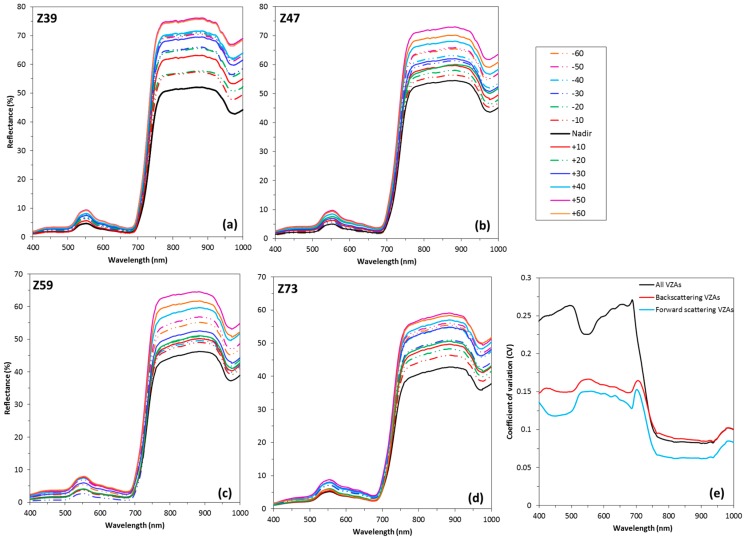
(**a**–**d**) Multi-angle spectral reflectance measured from −60° to +60° VZAs during stem elongation (Z39), booting (Z47), heading (Z59) and milk-filling (Z73) stages of wheat in 2007; and (**e**) curves of the coefficient of variation (CV) of reflectance measured at the booting stage (Z47) among the backscattering VZAs (from +10° to +60°), forward scattering VZAs (from −10° to −60°) and all thirteen VZAs.

The spectral reflectance measured from −60° to +60° VZAs showed considerable variation (Figure 3a–d). The patterns of the canopy spectra at different growth stages were very similar. It can be observed that reflectance values acquired from backscattering viewing angles were generally larger than those from corresponding nadir and forward scattering angles. +50° viewing angle produced the highest reflectance throughout the range of wavelengths studied (400–1000 nm), whereas the weakest reflectance values were obtained near the nadir to −20° angles in the visible region and the nadir direction in the NIR. At the backscattering directions, from +10° to +50° angles, reflectance increased with increasing VZAs. The result indicated that the sensor at 50° backscattering direction, close to the sun zenith angle, i.e., the “hotspot”, collected more reflected radiation than when placed at the other view angles.

To compare differences of canopy reflectance over the spectral range from visible to NIR (400–1000 nm) among VZAs, we calculated the coefficient of variation (CV) of spectral reflectance among the six backscattering VZAs, the six forward scattering VZAs and all thirteen VZAs, respectively. The curves calculated at the booting stage (Z47) were used as an example (Figure 3e), due to the similar patterns among growth stages. From the curve of all VZAs, it appears that canopy reflectance in visible bands yielded higher CVs than for the red edge and NIR bands, with two peaks in the red (650–680 nm) and then the blue (480–510 nm) regions. In contrast, reflectance at wavelengths higher than 760 nm showed the lowest CVs, followed by the green region centered at 530 nm, which generated two troughs in the curve. However, the other two curves of CV showed that the positions of two peaks and two troughs in visible to red edge bands were reversed, with two peaks in the green and red edge bands and two troughs in the blue and red bands, implying that the differences of reflectance in high Chl absorbing spectral bands were larger than that in scattering bands, when changing sensor viewing angles within back scattering or forward scattering directions.

### 3.3. Sensitivity Analyses of Published Spectral Indices Derived from VZAs Data to Leaf Chlorophyll Content in Vertical Layers

Given the different response of spectral wavebands at sensor viewing angles (Figure 3), the values of spectral indices developed on the band combinations of multi-angle spectral reflectance may be affected [44]. To explore the sensitivity of published spectral indices to the quantification of the vertical profile of leaf Chl, we analyzed the linear relationships between indices and Chl content at all VZAs for each leaf layer. As shown in Table 2, Table 3 and Table 4, the coefficients of determination (R^2^) in all three layers varied strongly with VZAs. Compared to back scattering observations, the performances of spectral indices at the corresponding forward scattering angles were poor. The differences of R^2^ values between the two directions ranged from 0.02 to 0.70. For this reason, the sensitivity of indices at the backscattering viewing angles and the nadir was analyzed in more detail. In the upper leaf layer, the R^2^ for most of the spectral indices increased generally with increasing backscattering VZAs and achieved maximum values at +50° viewing angle, followed by the nadir direction (Table 2). The best index was the CI_green_ at +50° viewing angle, which explained 70% of the variation in upper-layer Chl content. In the middle-layer of leaves, the R^2^ values of spectral indices first increased and then decreased in the backscattering direction and the highest R^2^ values were obtained at +30° observation (Table 3). Interestingly, in contrast to the upper-layer, leaf Chl content in the middle-layer correlated better with indices that use bands in red to red edge and NIR than in green and NIR, such as NDVI, PSNDa and CI_red edge1_ at +30° angle, which showed higher coefficients of determination (R^2^ = 0.75, 0.72 and 0.69, respectively). Two SR-like indices, PSSRa and PSSRb, yielded good relationships with leaf Chl content as well. It is worth to note that in the nadir direction, almost all spectral indices provided stronger relationships with leaf Chl content in the upper-layer, whereas they showed poor relationships with leaf Chl in the bottom-layer (R^2^ ≤ 0.50). This may present convincing evidence that the upper leaf layer contributed more to the nadir spectral reflectance, and spectral indices based on the nadir measurement tended to be less sensitive to Chl content in lower layer. However, there were higher R^2^ values for leaf Chl estimation in the bottom-layer, when indices were calculated from reflectance collected at +20° viewing angle, except for PRI, MCARI and TCARI. The strongest correlations appeared with PSSRa (R^2^ = 0.58), which performed even better than the published three-band CI-like indices (Table 4).

### 3.4. Optimization of Two-Band and Three-Band Indices for Estimation of Leaf Chlorophyll Content in Vertical Layers

**Figure 4:**
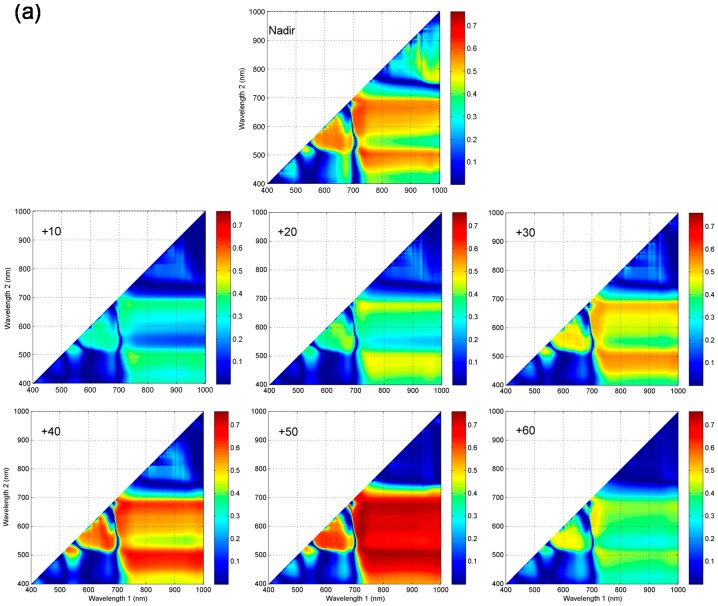

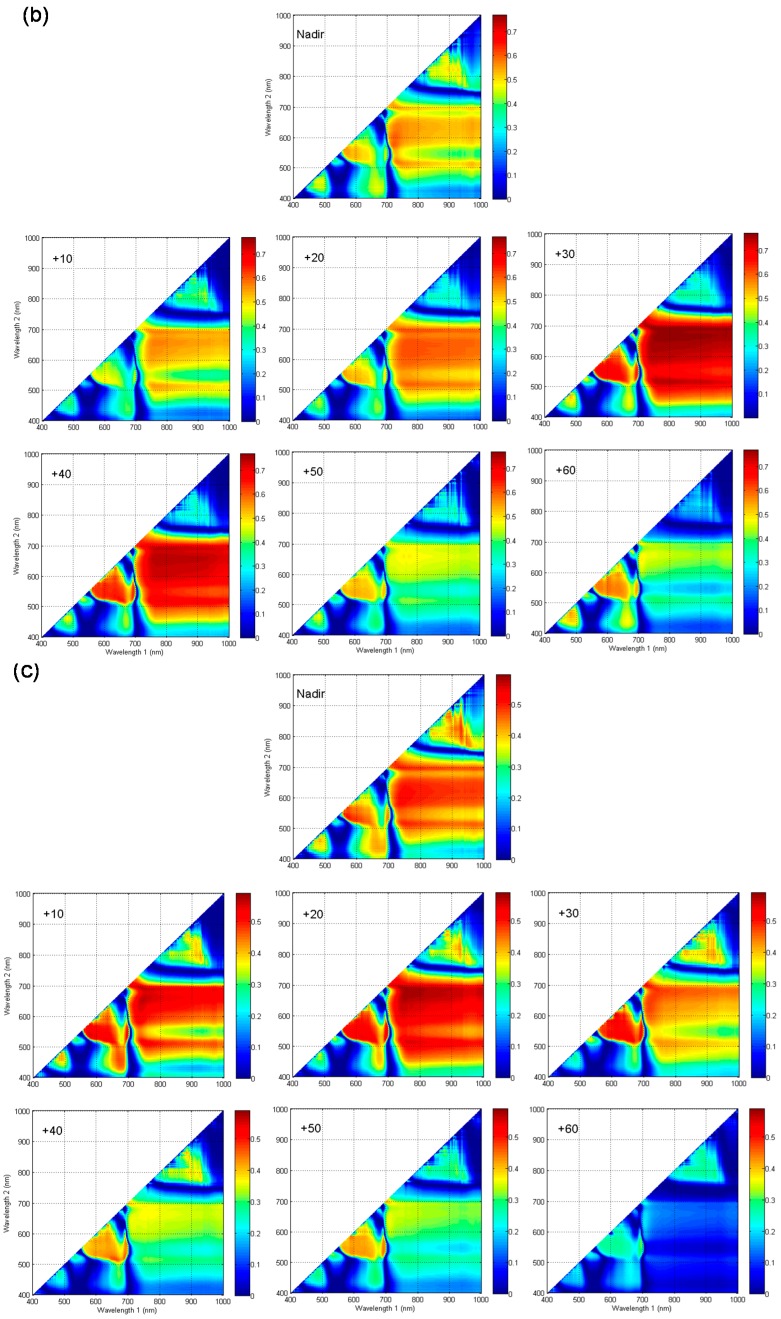
Contour maps of the coefficients of determination (R^2^) for the relationships between NDVI-like(*λ*1, *λ*2) indices (*λ*1 and *λ*2 are wavelength 1 and wavelength 2 on the corresponding axes) calculated from all possible two-band combinations from 400 to 1000 nm and leaf Chl content in: (**a**) the upper-layer; (**b**) the middle-layer; and (**c**) the bottom-layer of wheat canopies at the nadir and six backscattering viewing angles, respectively.

Since the band selection for Chl-related spectral indices listed in Table 1 was implemented by involving spectral bands from the blue, green, red, red edge and NIR regions and indices in SR-like, NDVI-like and CI-like types correlated well with vertical leaf Chl content, we examined all the possible two-band combinations from 400 nm to 1000 nm in the NDVI- and SR-like types at the nadir and all back scattering directions. These combinations were correlated with leaf Chl content in the three layers of wheat canopy. The sensitivity of different band combinations to leaf Chl was indicated by the values of R^2^. Figure 4 shows the R^2^ contour maps for NDVI-like type of indices relating to vertical leaf Chl content at different VZAs. The R^2^ contour maps for SR-like type of indices are shown in Figure S1. Both types of indices showed almost the same patterns at the corresponding viewing angles for each vertical layer. Similar to the results obtained with published spectral indices (Section 3.3), the +50°, nadir and +40° angles produced greater R^2^ than the other angles in leaf upper-layer Chl estimation, +30° and +40° angles, +20° and +10° angles outperformed in the estimation of leaf Chl in the middle and bottom layers. The best performance for NDVI-like and SR-like indices relating to Chl content in the above three vertical layers appeared in +50°, +30° and +20° viewing angles, respectively.

For a given canopy layer, the patterns of hot zones with higher R^2^ between two-band combinations in NDVI- and SR-like types and leaf Chl content were very similar among the optimal three or two viewing angles. However, in comparison, they varied greatly among the three vertical layers. The best NDVI- and SR-like indices relating to upper leaf Chl content were concentrated in three spectral regions: the shorter green bands (470–530 nm) paired with the NIR bands (740–1000 nm), and the bands from green to red bands (530–650 nm) paired with the red-edge bands (710–740 nm), and the red bands (650–680 nm) paired with the NIR bands (740–1000 nm) (Figure 4a and Figure S1a). The shorter green-NIR combinations also provided good results in the other two layers. However, they were relatively narrower, as compared to what was shown for the upper-layer (500–520 nm paired with 740–1000 nm in the middle-layer and 500–520 nm paired with 740–850 nm in the bottom-layer). In addition, the main and wider hot zone, which was dominant in determining leaf Chl content in the two lower layers, was the red to red edge bands paired with the NIR bands: 590–700 nm paired with 740–1000 nm for middle leaf Chl, whereas 620–700 nm paired with 740–1000 nm for bottom leaf Chl estimation (Figure 4b,c and Figure S1b,c).

**Figure 5:**
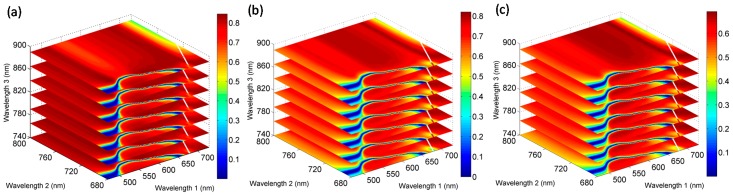
The three-dimensional slice maps of the coefficients of determination (R^2^) for the relationships between CI-like(*λ*1, *λ*2, *λ*3) indices calculated from all possible three-band combinations in the regions of 470–730 nm (*λ*1, Wavelength 1), 680–800 nm (*λ*2, Wavelength 2) and 740–1000 nm (*λ*3, Wavelength 3) and leaf Chl in: (**a**) the upper-layer; (**b**) the middle-layer; and (**c**) the bottom-layer at +50°, +30° and +20° viewing angles, respectively.

**Figure 6:**
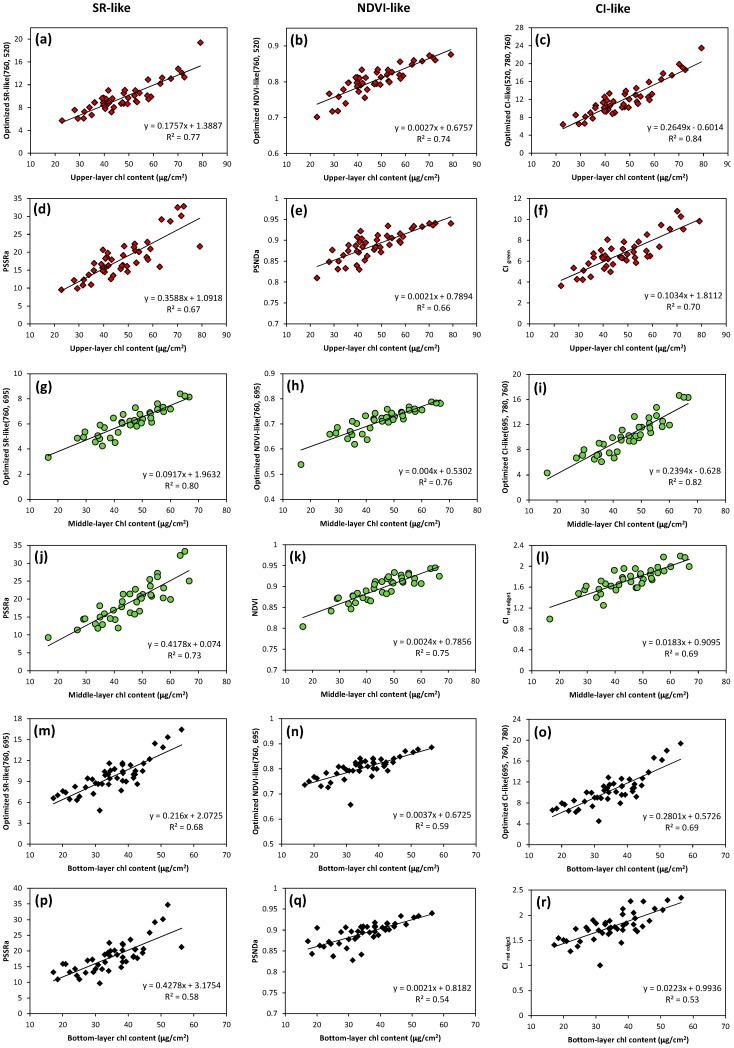
Scatter plots of the relationships between optimized SR-, NDVI- and CI-like types of indices and corresponding types of best performing published indices, versus leaf Chl content in: the upper-layer (**a**–**f**, red diamonds); the middle-layer (**g**–**l**, green circles); and the bottom-layer (**m**–**r**, black diamonds) of wheat canopies.

Based on the analyses presented above, we calculated coefficients of determination for the relationships between vertical leaf Chl content and the CI-like indices calculated from all possible three-band combinations in the regions of the shorter green to red edge bands (470–730 nm), the red to NIR bands (680–800 nm) and the NIR bands (740–1000 nm) at the nadir and backscattering observations. Due to the small differences in the patterns of sensitive spectral band combinations among viewing angles for the same layer, further analyses of spectral indices optimization were conducted only at the best angle for each vertical layer. Figure 5 shows the three-dimensional slice maps of R^2^ for the estimation of leaf Chl in the upper-, middle- and bottom-layer at +50°, +30° and +20° viewing angles, respectively. Three types of optimized indices composed of the central spectral bands in the zones with the highest R^2^ were selected and compared with the corresponding types of best performing published spectral indices (Table 2, Table 3 and Table 4), in the Chl estimation for each vertical layer. The scatter plots of the relationships are shown in Figure 6. In the upper-layer of leaves, the NDVI-like type of indices tended to saturate when Chl content exceeds 63 µg/cm^2^. However, the three-band optimized CI-like index (using 520, 780 and 760 nm wavebands) was more effective than the two-band spectral indices, explaining 84% of variation in leaf Chl content (Figure 6c). In the lower layers of leaves, all three types of optimized indices exploited the red edge and NIR bands, i.e., 695 nm paired with 760 and 780 nm. The optimized CI-like (695, 780, 760) index was linearly related to leaf Chl in the middle-layer and produced the highest R^2^ value of 0.82 (Figure 6i), whereas it did not result in a significant difference with optimized two-band indices in R^2^ (Figure 6g,h). For the bottom leaf Chl dataset, quite different results were observed compared to the other layers. The optimized CI-like (695, 760, 780) and SR-like (760, 695) indices showed little sensitivity to low leaf Chl content (Chl content < 27 µg/cm^2^) and yielded moderate correlations (R^2^ < 0.70) (Figure 6m,o), although they were still superior to the optimized NDVI-like (760, 695) index (Figure 6n). Figure 6 reveals that the three types of optimized spectral indices achieved higher R^2^ compared to published indices that have analogous forms, greatly improving the accuracy of estimation of leaf Chl content in each vertical layer within the canopy. Particularly, the three optimized CI-like indices yielded a significant increase in R^2^ by 20%, 19% and 30%, respectively, as compared to their published analogues, in modeling leaf Chl content from the top to the bottom layers.

### 3.5. Validation of Vertical Leaf Chlorophyll Content Estimation Models

**Figure 7:**
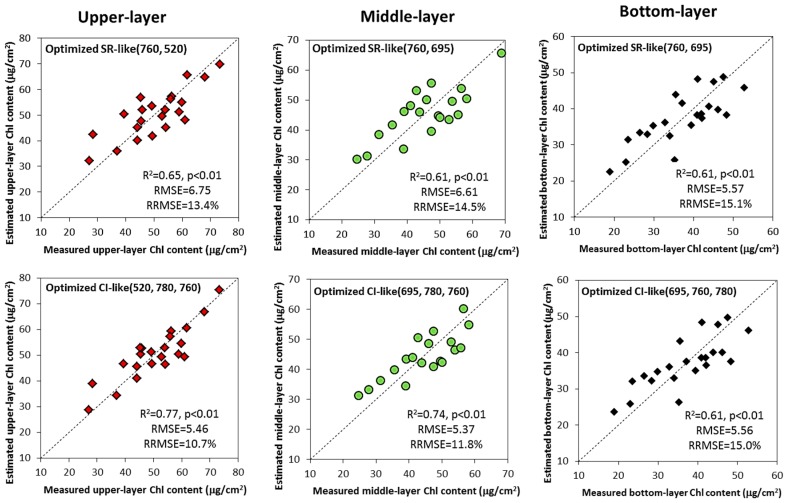
The comparison of validation models between the measured and estimated Chl content using the optimized spectral indices in the upper-layer (red diamonds), middle-layer (green circles) and bottom-layer (black diamonds), respectively. The predictive R^2^, *p*-value, RMSE and RRMSE shown refer to the validation dataset. Dotted lines are 1:1 lines.

Figure 7 shows the comparison of validation models between the measured and estimated Chl content in the three vertical layers using the optimized spectral indices based on the optimal viewing angle reflectance measurements (i.e., +50°, +30°, +20° for layers from the top to the bottom). In each layer, the two best types of optimized spectral indices were selected. Optimized CI-like (520, 780, 760) index produced the most accurate prediction of the upper-layer leaf Chl content, with R^2^ of 0.77 (*p* < 0.01) and RMSE value of 5.46 µg/cm^2^. The data points were scattered near the 1:1 line, which confirmed a consistent agreement between leaf Chl content measured in the field and those estimated by the optimized CI-like index. In the middle-layer, the model based on optimized CI-like (695, 780, 760) index also showed quite promising ability of prediction, with higher R^2^ than optimized SR-like (760, 695). For leaves in the bottom-layer, the optimized SR-like (760, 695) and CI-like (695, 760, 780) indices generated comparable results, with R^2^ of 0.61 (*p* < 0.01) and RMSE of 5.57 µg/cm^2^ for the SR-like type index and R^2^ of 0.61 (*p* < 0.01) and RMSE of 5.56 µg/cm^2^ for CI-like type index. However, both overestimated leaf Chl at low values (Chl < 30 µg/cm^2^). In addition, RRMSE values suggested higher prediction accuracy with the two types of optimized spectral indices in the upper and middle layers than that in the bottom-layer. 

## 4. Discussion

Leaf Chl content has a characteristic vertical heterogeneity within winter wheat canopies, showing top-down declining trends along the canopy depth at growth stages from stem elongation to milk-filling (Figure 2). It is important to consider this vertical heterogeneity, when attempting to remotely assess the real nutritional status of crops. The purpose of our study was to explore the mechanisms of estimation of leaf Chl content in vertical layers using multi-angle hyperspectral data. Measurements of multi-angle hyperspectral reflectance in situ revealed an increased reflectance in the backscattering observations, but lower reflectance in the forward scattering directions (Figure 3a–d), which is consistent with the typical bidirectional reflectance distribution function (BRDF) behavior [45,46]. Moreover, all spectral indices in the backscattering observations were more efficient in leaf Chl estimation for each vertical layer than those in the forward scattering reflectance (Table 2, Table 3 and Table 4). These are explained by the fact that, in the backscattering directions, the sensor views a higher amount of sunlit leaves, while the forward scattering directions contain far more signal from shaded leaves and few photons could escape from the canopy [20,47], resulting in the difficulty of capturing the information on leaf Chl in these directions. Nevertheless, the magnitude of the differences between the backscattering and forward scattering directions in leaf Chl estimation is not fully understood yet. Our results are consistent with the findings of other researchers, who reported that backscattering directions served much better than forward scattering directions in the estimation of vegetation parameters [48,49,50].

The vertical profile of leaf Chl content within canopy can be detected by multi-angle spectral reflectance measured from the nadir and backscattering viewing angles. This is because the proportions of canopy components in the upper-, middle- and bottom-layer viewed by a sensor change when measured from different VZAs [21,51]. However, understanding which canopy layer the sensor is seeing from a certain viewing angle should be addressed first. Huang et al. [21] identified the dominant vertical layer of leaves in digital camera pictures of wheat canopies, which corresponded to the same angles as spectral reflectance measurements, and then determined the most sensitive viewing angles for estimation of Chl content in each layer. It is a qualitative method, but needs to be quantitatively validated with experimental data. In this study, we analyzed the correlations between leaf Chl content and published Chl-related spectral indices as well as all possible combinations over spectral bands in NDVI-, SR- and CI-like types of indices at all viewing angles for each vertical layer, systematically. The results suggest that viewing angles of +50°, nadir and +40° can be selected to observe leaves of the upper-layer, +30° and +40° of the middle-layer, and +20° and +10° of the bottom-layer, for Chl estimation. These results depend on the geometry of observation employed in the present study, but might provide more general indications. View angles close to the hotspot are more suitable for top of the canopy Chl estimation, whereas smaller off-nadir view zenith angles allow a better assessment of bottom of the canopy Chl. Leaves in the upper-layer of canopy receive a larger solar irradiance, thus a larger amount of radiance is scattered towards the sensor than from leaves in the bottom-layer. With increasing backscattering VZAs, the proportion of sunlit canopy components in the upper-layer viewed by the sensor continuously increases, hence R^2^ values between spectral indices and upper leaf Chl content increased from +10° to +50° (Table 2). However, R^2^ values decreased when the sensor kept moving to larger angles (i.e., +60°). The fraction of sunlit leaves viewed from the backscattering directions first increases from nadir, but then it decreases at angles exceeding the sun zenith angle [52]. It can be expected that the sunlit leaves fraction viewed differs for different canopy layer, as a consequence of the masking of canopy elements. In particular, for the upper canopy layer, an increasing amount of wheat spikes, rather than Chl containing leaves, is included in the field of view of the sensor at these angles, during late growth stages of wheat (heading to milk-ripe stages). This affects the canopy spectral reflectance [6,15] leading to poorer performances of spectral indices for upper leaf Chl estimation at these angles.

Although a variety of spectral indices were developed to estimate leaf Chl content in the literature [31,37,39], to our knowledge, few researchers investigated the sensitivity of these indices to leaf Chl content in different vertical layers in current studies. Our work shows that at the nadir observation, relatively little information on Chl in the lower layer of canopies can be obtained from nadir measurements (Table 2, Table 3 and Table 4). This result also indirectly confirmed what was found by Xiao et al. [53], who reported that there is no apparent influence on whole canopy reflectance, when the lower leaf layers of wheat are removed.

The calculations of all possible two-band and three-band combinations over spectral bands in NDVI-, SR- and CI-like types of indices, carried out in this study, was aimed at a systematical search for the most sensitive band combinations and optimized two-band and three-band spectral indices for estimating vertical leaf Chl content. Our result indicates that sensitive band combinations relating to leaf Chl for vertical layers were different. This is why the predictive ability of published spectral indices that have analogous forms, but varying band combinations, was different when assessing leaf Chl content in different layers. For instance, the CI_green_ index performed better than the CI_red edge1_ in the upper-layer, while the latter was superior to the former in the middle-layer at all VZAs (Table 2 and Table 3). In the studies of Stagakis et al. [27] and He et al. [46], the patterns of hot zones for the relationships between leaf Chl or nitrogen content and SR- and NDVI-like types of indices varied depending on the VZA. This did not agree with our result, in part probably because they considered the whole plant canopy without considering non-uniform vertical characteristics of biochemical parameters. Due to the small differences among viewing angles, in a given vertical layer, we could choose the same spectral index, or even assemble information from several desired viewing angles, in order to estimate leaf Chl content in the future. 

The spectral bands selected for the optimized SR-, NDVI- and CI-like types of indices were the shorter green, red edge and NIR bands, i.e., center around 520 nm, 695 nm, 760 nm and 780 nm, respectively (Figure 6). A high sensitivity of reflectance in the green and red edge regions and very low sensitivity in NIR to Chl level has been highlighted in earlier studies [14,54,55], thus the optimized spectral indices they constructed were suitable to estimate leaf Chl content. In the upper leaf Chl estimation, the shorter green band improved the performance of spectral indices when combined with NIR bands. However, the red edge and NIR bands were the best band combination for leaf Chl estimation in the two lower canopy layers. This is not consistent with the results of Huang et al. [21], who used the same spectral index to estimate leaf Chl content in different vertical layers. Considering the leaf stacking effect, in the visible spectral region strong absorption is dominated by leaf Chl in the upper layer, but with an exponential attenuation of light with canopy depth, according to the Beer–Lambert Law. Conversely, in the long wave part of the red edge, the absorbance is relatively small, resulting in an increasing scattering. Because attenuation with depth by scattering is weaker than by absorption [25], a larger amount of light penetrates to and is backscattered from the lower layers. In addition, the light in the red edge bands has proved to have much deeper penetration inside the canopy than in the green bands [26]. Optimized spectral indices that combined the red edge and NIR bands in our study, therefore, were applicable to estimate leaf Chl content in the lower layers, rather than the same green spectral indices optimized in the upper leaf Chl assessment. However, relatively higher proportion of intercanopy shadow, soil background and non-photosynthetic materials within the field of view in the bottom-layer makes it difficult to derive the maximum leaf Chl information using the optimized spectral indices, leading to lower estimation accuracy compared to the other two layers (Figure 7). Gitelson et al. [56] and Wang et al. [18] found that two-band spectral indices often constrain the regression analysis and that multiple hyperspectral narrow bands can provide additional information and were more robust to estimate vegetation variables of interest. However, our results show similarities and differences with such finding, depending on the vertical layers. For example, the three-band optimized CI-like type index improved the saturation problem existing in NDVI-like type indices, thereby achieved the highest significant in relation to leaf Chl content in the upper-layer, whereas it showed less sensitivity to low Chl content in the bottom layer. Due to the comparable predictive abilities of optimized CI- and SR-like indices in the bottom-layer, we suggest that if a third band is not available, two-band SR-like indices are good option in bottom leaf Chl estimation. To our knowledge, this is the first study to calculate all possible three-band combinations in CI-like index type and investigate their performances in vertical leaf Chl assessment within wheat canopies. Our results show the importance of accounting for the sensitivity of spectral bands and penetration characteristics of the light in the selected spectral region inside the canopy, as well as the formula that was used to derive the spectral index when estimating vertical profile of leaf Chl within a wheat canopy.

Our study provides support for the selection of spectral wavebands and viewing angles to design effective ground-based proximal sensors, and even for further research on the detection of leaf Chl content in different vertical layers of crop canopies, based on airborne off-nadir imaging spectroscopy. Due to the strong correlation between leaf Chl and N content [10], our study contributes to the development of methods for the assessment of the vertical distribution of N status, which can reflect precise nutritional condition of plant and offer scientific guidance for agricultural management [24]. Another important possible application of this study is to provide vertical profile of leaf Chl content for the calibration or validation of multiple-layer canopy radiative transfer models (MRTM), allowing to simulate canopy spectral reflectance more accurately and reduce uncertainty of the retrievals. Given that data were collected at different wheat growth stages in different years and a limited number of samples was available in each growth stage in this study, a large uncertainty would have been introduced if the samples had been split into subsets according to different growth stages, assuming separate models of vertical profile of leaf Chl content. Moreover, canopy multi-angle hyperspectral reflectance contains comprehensive information on the structure and biochemistry of plant canopy, developing a Chl-related index that is not influenced by other canopy biophysical variables (e.g., LAI) has been the aim of many studies [1,9,14]. Further investigation is required to study the effects of crop growth stages and vertical distribution of LAI on an effective vertical leaf Chl monitoring. Although optimized SR-, NDVI- and CI-like indices increased the R^2^ relating to leaf Chl in each layer compared to the published indices (Figure 6), they were optimized under a relatively limited number of samples, and all wheat cultivars used were erectophile-type and for a specific agroecological condition. These new indices need to be further tested by more experimental data, different cultivar types (e.g., planophile-type) and in other ecosystems.

## 5. Conclusions

This study shows that reflectance measured from off-nadir observations holds more promising potential in estimating vertical profiles of leaf Chl content than that from the nadir observation where relatively little information on Chl in the lower canopy layers can be detected. Viewing angles close to the hotspot are more suitable for the upper Chl estimation, whereas smaller off-nadir viewing angles achieve more accurate assessment of Chl in the lower canopy layers. The best spectral band combinations for the upper-layer were green paired with NIR bands, whereas red edge paired with NIR bands were better for the lower layers, indicating that leaf stacking effect and the penetration characteristics inside the canopy of the light in the Chl absorption spectral regions have impacts on the estimation of the vertical distribution of leaf Chl. In addition, the formula of the spectral indices is another factor. The three-band optimized CI-like types of indices achieved better performance in quantifying leaf Chl in the upper and middle layers. However, they showed less sensitivity to low leaf Chl content in the bottom layer, for which the two-band optimized SR-like index was more appropriate. This study provides insights into remote estimation of vertical distribution of leaf Chl content within canopies, allowing more accurate monitoring of crop real nutritional status and providing practical guidance for the design of optimized proximal sensors, e.g., to support precision nitrogen fertilization.

## Figures and Tables

**Table 1:** Published spectral indices tested in this study. Indices were sorted according to their band number.

Spectral Indices	Formula	Reference
*Two-band spectral indices*		
PSSRa (Pigment specific simple ratio)	$R_{800}/R_{680}$	[31]
PSSRb (Pigment specific simple ratio)	$R_{800}/R_{635}$	[31]
PSNDa (Pigment specific normalized difference)	$\left(R_{800}-R_{680}\right)/(R_{800}+R_{680})$	[31]
PSNDb (Pigment specific normalized difference)	$\left(R_{800}-R_{635}\right)/(R_{800}+R_{635})$	[31]
GI (Green index)	$R_{554}/R_{677}$	[32]
PRI (Photochemical reflectance index)	$\left(R_{531}-R_{570}\right)/(R_{531}+R_{570})$	[33]
NDVI (Normalized difference vegetation index)	$\left(R_{800}-R_{670}\right)/(R_{800}+R_{670})$	[34]
NDVI2 (Normalized difference vegetation index)	$\left(R_{750}-R_{705}\right)/(R_{750}+R_{705})$	[35]
*Three-band spectral indices*		
MCARI (Modified chlorophyll absorption ratio index)	$\left[(R_{700}-R_{670})-0.2(R_{700}-R_{550})](R_{700}/R_{670}\right)$	[36]
TCARI (Transformed chlorophyll absorption ratio index)	$3[(R_{700}-R_{670})-0.2(R_{700}-R_{550})(R_{700}/R_{670})]$	[1]
MTCI (MERIS Terrestrial Chlorophyll index)	$\left(R_{754}-R_{709}\right)/(R_{709}-R_{681})$	[37]
CI_green_ (Chlorophyll index at green band)	$(R_{550}^{-1}-R_{840–870}^{-1})\times R_{840–870}$	[38,39]
CI_red edeg1_ (Chlorophyll index at red edge band)	$(R_{695–740}^{-1}-R_{750–800}^{-1})\times R_{750–800}$	[38,39]
CI_red edge2_ (Chlorophyll index at red edge band)	$(R_{720–730}^{-1}-R_{840–870}^{-1})\times R_{840–870}$	[38,39]
SIPI (Structure-insensitive pigment index)	$\left(R_{800}-R_{445}\right)/(R_{800}-R_{680})$	[40]

**Table 2:** Coefficients of determination (R^2^) between published spectral indices and leaf Chl content in the upper-layer of wheat canopy at different viewing angles.

	−60	−50	−40	−30	−20	−10	Nadir	+10	+20	+30	+40	+50	+60
*Two-band indices*													
PSSRa	0.15	0.14	0.03	0.03	0.06	0.14	0.60	0.27	0.32	0.28	0.51	0.67	0.27
PSSRb	0.19	0.16	0.07	0.04	0.09	0.16	0.62	0.34	0.39	0.31	0.46	0.67	0.27
PSNDa	0.17	0.11	0.05	0.02	0.09	0.12	0.56	0.36	0.45	0.33	0.61	0.66	0.41
PSNDb	0.16	0.10	0.02	0.01	0.04	0.08	0.54	0.27	0.33	0.27	0.46	0.63	0.32
GI	0.19	0.19	0.16	0.11	0.19	0.20	0.51	0.26	0.33	0.27	0.46	0.41	0.32
PRI	0.23	0.18	0.07	0.07	0.08	0.10	0.42	0.09	0.11	0.14	0.20	0.37	0.29
NDVI	0.16	0.11	0.04	0.01	0.06	0.10	0.57	0.33	0.41	0.31	0.53	0.65	0.35
NDVI2	0.10	0.06	0.00	0.00	0.01	0.06	0.43	0.17	0.26	0.22	0.35	0.56	0.24
*Three-band indices*													
MCARI	0.02	0.02	0.09	0.10	0.13	0.08	0.04	0.06	0.00	0.00	0.00	0.01	0.02
TCARI	0.00	0.00	0.04	0.08	0.10	0.05	0.00	0.02	0.00	0.00	0.04	0.12	0.07
MTCI	0.07	0.03	0.00	0.00	0.00	0.03	0.36	0.03	0.11	0.12	0.17	0.42	0.15
CI_green_	0.09	0.04	0.00	0.00	0.00	0.05	0.62	0.12	0.26	0.21	0.35	0.70	0.19
CI_red edeg1_	0.09	0.04	0.01	0.00	0.00	0.05	0.49	0.08	0.21	0.19	0.28	0.64	0.18
CI_red edge2_	0.07	0.02	0.00	0.00	0.00	0.03	0.30	0.03	0.14	0.12	0.19	0.43	0.14
SIPI	0.12	0.12	0.05	0.04	0.12	0.09	0.39	0.18	0.18	0.15	0.18	0.24	0.19

**Table 3:** Coefficients of determination (R^2^) between published spectral indices and leaf Chl content in the middle-layer of wheat canopy at different viewing angles.

	−60	−50	−40	−30	−20	−10	Nadir	+10	+20	+30	+40	+50	+60
*Two-band indices*													
PSSRa	0.08	0.18	0.15	0.22	0.34	0.40	0.52	0.47	0.51	0.73	0.57	0.34	0.14
PSSRb	0.10	0.21	0.19	0.22	0.34	0.35	0.48	0.49	0.53	0.70	0.55	0.32	0.14
PSNDa	0.23	0.22	0.18	0.20	0.36	0.37	0.49	0.55	0.58	0.72	0.62	0.46	0.41
PSNDb	0.19	0.19	0.17	0.22	0.33	0.39	0.57	0.50	0.56	0.70	0.61	0.41	0.38
GI	0.29	0.29	0.26	0.26	0.31	0.32	0.35	0.22	0.25	0.49	0.37	0.31	0.32
PRI	0.40	0.29	0.32	0.32	0.38	0.40	0.48	0.32	0.39	0.56	0.42	0.42	0.41
NDVI	0.22	0.22	0.18	0.20	0.35	0.36	0.50	0.50	0.54	0.75	0.61	0.41	0.38
NDVI2	0.07	0.10	0.09	0.14	0.23	0.28	0.49	0.43	0.53	0.70	0.58	0.36	0.26
*Three-band indices*													
MCARI	0.09	0.07	0.07	0.11	0.12	0.04	0.03	0.02	0.01	0.00	0.01	0.00	0.02
TCARI	0.03	0.01	0.02	0.04	0.05	0.01	0.03	0.00	0.04	0.01	0.07	0.03	0.07
MTCI	0.01	0.03	0.02	0.06	0.10	0.24	0.30	0.20	0.39	0.47	0.42	0.22	0.09
CI_green_	0.02	0.05	0.02	0.07	0.15	0.30	0.37	0.29	0.43	0.60	0.51	0.23	0.08
CI_red edeg1_	0.03	0.08	0.05	0.11	0.18	0.30	0.40	0.32	0.50	0.69	0.55	0.29	0.13
CI_red edge2_	0.01	0.04	0.02	0.06	0.11	0.21	0.29	0.16	0.38	0.43	0.41	0.20	0.08
SIPI	0.36	0.24	0.31	0.21	0.40	0.39	0.40	0.42	0.43	0.49	0.45	0.37	0.40

**Table 4:** Coefficients of determination (R^2^) between published spectral indices and leaf Chl content in the bottom-layer of wheat canopy at different viewing angles.

	−60	−50	−40	−30	−20	−10	Nadir	+10	+20	+30	+40	+50	+60
*Two-band indices*													
PSSRa	0.04	0.14	0.19	0.24	0.32	0.35	0.50	0.53	0.58	0.47	0.29	0.24	0.01
PSSRb	0.06	0.20	0.28	0.26	0.33	0.32	0.46	0.53	0.56	0.49	0.29	0.22	0.01
PSNDa	0.08	0.09	0.19	0.17	0.27	0.31	0.43	0.54	0.54	0.47	0.36	0.33	0.13
PSNDb	0.05	0.05	0.12	0.15	0.22	0.29	0.50	0.46	0.51	0.40	0.26	0.26	0.09
GI	0.21	0.37	0.37	0.31	0.27	0.25	0.28	0.28	0.31	0.39	0.25	0.24	0.14
PRI	0.17	0.18	0.21	0.27	0.29	0.33	0.49	0.31	0.41	0.36	0.25	0.28	0.14
NDVI	0.06	0.08	0.17	0.15	0.23	0.27	0.44	0.47	0.53	0.43	0.27	0.26	0.10
NDVI2	0.01	0.01	0.04	0.09	0.17	0.29	0.47	0.50	0.52	0.35	0.24	0.21	0.04
*Three-band indices*													
MCARI	0.14	0.21	0.17	0.17	0.11	0.02	0.00	0.05	0.00	0.00	0.03	0.02	0.04
TCARI	0.08	0.10	0.06	0.08	0.04	0.00	0.01	0.01	0.00	0.02	0.01	0.00	0.02
MTCI	0.00	0.00	0.01	0.04	0.11	0.26	0.33	0.28	0.39	0.32	0.16	0.13	0.00
CI_green_	0.00	0.01	0.03	0.07	0.15	0.30	0.35	0.39	0.48	0.34	0.20	0.14	0.00
CI_red edge1_	0.00	0.01	0.04	0.08	0.17	0.31	0.38	0.45	0.53	0.40	0.24	0.18	0.01
CI_red edge2_	0.00	0.00	0.02	0.04	0.11	0.24	0.30	0.33	0.39	0.31	0.15	0.11	0.00
SIPI	0.10	0.08	0.12	0.11	0.21	0.24	0.37	0.43	0.37	0.36	0.23	0.23	0.14

## Supplementary Materials

The following are available online at http://www.mdpi.com/1424-8220/17/12/2711/s1, Figure S1: Contour maps of the coefficients of determination (R^2^) for the relationships between SR-like(*λ*1, *λ*2) indices (*λ*1 and *λ*2 are wavelength 1 and wavelength 2 on the corresponding axes) calculated from all possible two-band combinations from 400 to 1000 nm and leaf Chl content in: (a) the upper-layer; (b) the middle-layer; and (c) the bottom-layer of wheat canopies at the nadir and six backscattering viewing angles, respectively.
