# Supplementary material for: Off-Nadir Hyperspectral Sensing for Estimation of Vertical Profile of Leaf Chlorophyll Content within Wheat Canopies
Source: Sensors (Basel). 2017 Nov 23;17(12):2711. doi: 10.3390/s17122711 (PMC5751501; doi:10.3390/s17122711)
Supplement: Supplementary file 1 [file sensors-17-02711-s001.pdf]

Supplementary Materials: Off-Nadir Hyperspectral Sensing for Estimation of Vertical Profile of Leaf Chlorophyll Content within Wheat Canopies

Weiping Kong [1,2], Wenjiang Huang [1,3,*], Raffaele Casa [4], Xianfeng Zhou [1], Huichun Ye [1] and Yingying Dong [1]

[1] Key Laboratory of Digital Earth Science, Institute of Remote Sensing and Digital Earth, Chinese Academy of Sciences, Beijing 100094, China; kongwp@radi.ac.cn (W.K.); zhouxf@radi.ac.cn (X.Z.); yehuichun000@126.com (H.Y.); dongyy@radi.ac.cn (Y.D.)

[2] University of Chinese Academy of Sciences, Beijing 100049, China

[3] State Key Laboratory of Remote Sensing Science, Institute of Remote Sensing and Digital Earth, Chinese Academy of Sciences, Beijing 100094, China

[4] Department of Agricultural and Forestry scieNcEs (DAFNE), Università degli Studi della Tuscia, Via San Camillo de Lellis, 01100 Viterbo, Italy; rcasa@unitus.it

* Correspondence: huangwj@radi.ac.cn; Tel.: +86-10-82178169

Received: 14 September 2017; Accepted: 22 November 2017; Published: date

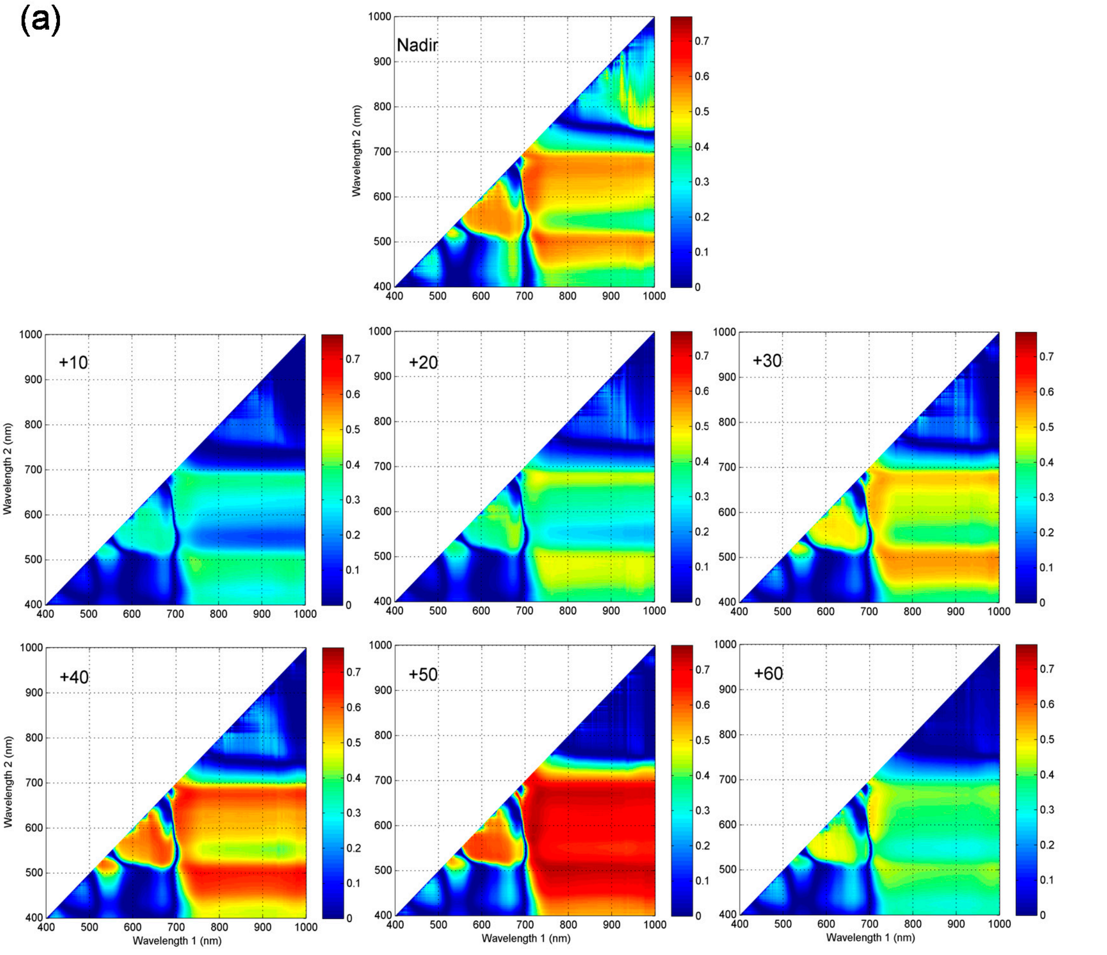

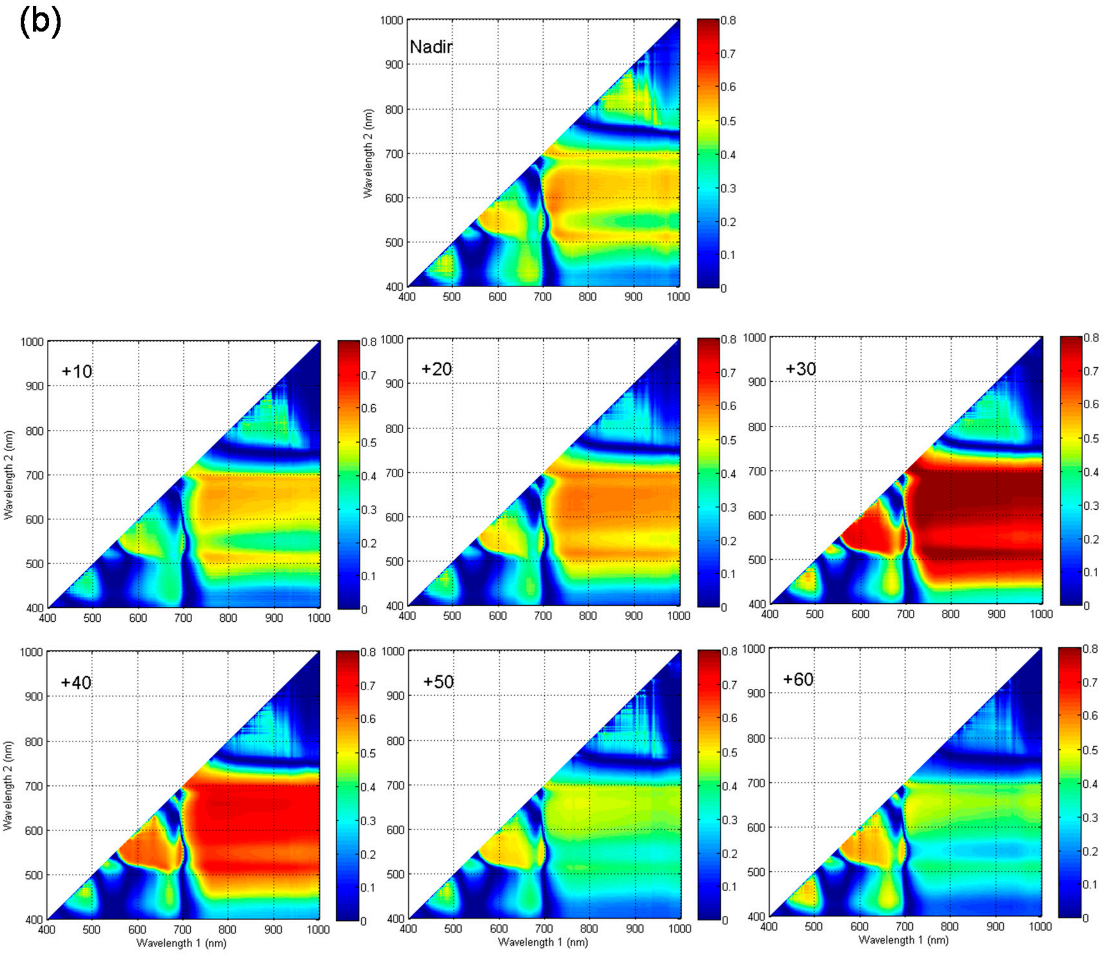

(b)
Nadir
+10
+20
+30
+40
+50
+60
Wavelength 1 (nm)
Wavelength 2 (nm)

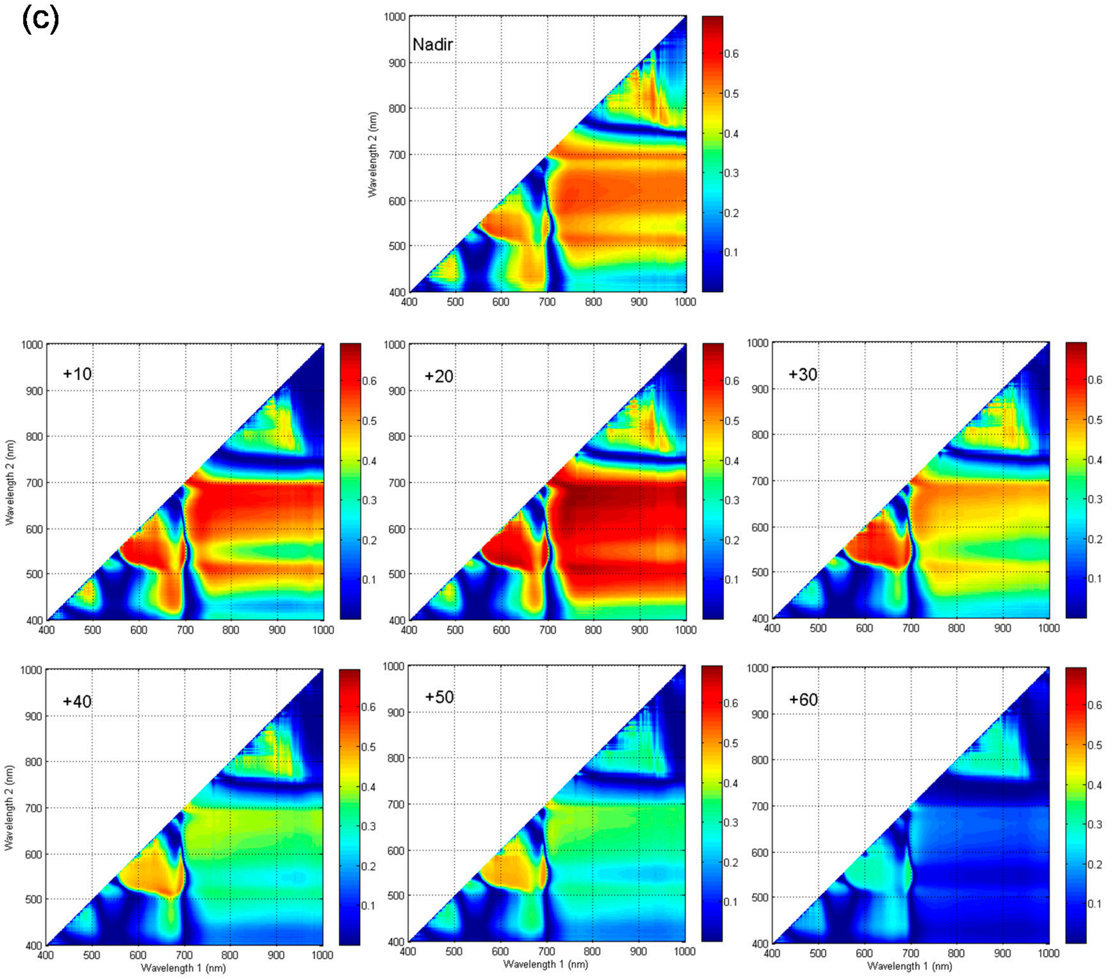

Figure S1. Contour maps of the coefficients of determination (R^2) for the relationships between SR-like($\lambda1$, $\lambda2$) indices ($\lambda1$ and $\lambda2$ are wavelength 1 and wavelength 2 on the corresponding axes) calculated from all possible two-band combinations from 400 to 1000 nm and leaf Chl content in (**a**) the upper-layer, (**b**) the middle-layer, and (**c**) the bottom-layer at the nadir and six backscattering viewing angles, respectively.
